# Mental Imagery and Acute Exercise on Episodic Memory Function

**DOI:** 10.3390/brainsci9090237

**Published:** 2019-09-18

**Authors:** Lauren Johnson, Jie Yao, Liye Zou, Tao Xiao, Paul D. Loprinzi

**Affiliations:** 1 Exercise & Memory Laboratory, Department of Health, Exercise Science and Recreation Management, The University of Mississippi, University, MS 38677, USA; lmjohns7@go.olemiss.edu (L.J.); pdloprin@olemiss.edu (P.D.L.); 2 School of Humanities and Social Sciences, Harbin Institute of Technology (Shenzhen), Shenzhen 518055, China; yaojiejulie@hit.edu.cn; 3 Exercise and Mental Health Laboratory, Shenzhen University, Shenzhen 518060, China; Liyezou123@gmail.com; 4 College of Mathematics and Statistics, Shenzhen University, Shenzhen 518060, China

**Keywords:** cognition, imagination, mental imagery, memory, physical activity

## Abstract

Mental imagery is used extensively in the sporting domain. It is used for performance-enhancement purposes, arousal regulation, affective and cognitive modification, and rehabilitation purposes. The purpose of this experiment was to evaluate whether acute exercise and mental imagery of acute exercise have similar effects on cognitive performance, specifically memory function. A within-subject randomized controlled experiment was employed. Participants (*N* = 24; M_age_ = 21.5 years) completed two exercise-related visits (i.e., actual exercise and mental imagery of exercise), in a counterbalanced order. The acute-exercise session involved 10 min of intermittent sprints. The mental-imagery session involved a time-matched period of mental imagery. After each manipulation (i.e., acute exercise or mental imagery of acute exercise), memory was evaluated from a paired-associative learning task and a comprehensive evaluation of memory, involving spatial–temporal integration (i.e., what, where, and when aspects of memory). Bayesian analyses were computed to evaluate the effects of actual exercise and mental imagery of exercise on memory function. For the paired-associative learning task, there was moderate evidence in favor of the null hypothesis for a main effect for condition (BF_01_ = 2.85) and time by condition interaction (BF_01_ = 3.30). Similarly, there was moderate evidence in favor of the null hypothesis for overall (what-where-when) memory integration (BF_01_ = 3.37), what-loop (BF_01_ = 2.34), where-loop (BF_01_ = 3.45), and when-loop (BF_01_ = 3.46). This experiment provides moderate evidence in support of the null hypothesis. That is, there was moderate evidence to support a non-differential effect of acute exercise and mental imagery of acute exercise on memory function.

## 1. Introduction

Mental imagery is used extensively in the sporting domain. It is used for performance-enhancement purposes, arousal regulation, affective and cognitive modification, and rehabilitation purposes [1]. Much less focus, however, has been given to investigating the effects of mental imagery on exercise-related cognitive outcomes that are not exclusively focused within the sporting domain but, importantly, have implications for both the exercise and sporting domains. That is, cognitive performance is critical for both sport performance and exercise behavior [2], with the latter behavior considered in a less-competitive context.

Acute exercise has been shown to enhance memory function for both episodic- and working-memory tasks [3,4,5]. As we detailed recently [6], higher-intensity exercise may be more beneficial for episodic memory tasks, when compared to working-memory tasks. Recent research also demonstrates that imagined exercise activates similar brain regions to those activated during actual engagement in exercise [7,8]. For example, the mental imagery of running has been shown to enhance parahippocampal and cerebellar neural activity, which take place in brain regions associated with memory function [9,10,11]. Further, a recent top-down model of voluntary mental imagery highlights three main steps involved in the mental-imagery process [12]. The creation of a mental image begins in the frontal cortex (step one), which triggers a cascade of neural events involved in retrieving stored information from more posterior regions, such as the medial temporal lobe (step two). Following this, sensory and spatial representations of the imagery are formed (step three). Notably, if other representations, such as movement and spatial locations, are called upon, then the middle temporal lobe and parietal lobe are activated [12]. As thoroughly detailed elsewhere [13], imagery of exercise may partially reactivate perceptual, motor, and introspective states that underlie past sensorimotor experience. 

To our knowledge, however, no study has evaluated whether actual exercise engagement vs. mental imagery of acute exercise have similar effects on episodic memory function, which was the purpose of this study, written as a brief report. As stated, such an effect is plausible, as both actual and imagined exercise activate similar brain structures. This is a worthwhile research endeavor for several reasons. If both acute exercise and mental imagery of exercise have similar effects on memory, then this will help shed light on the potential overlapping mechanisms subserving memory. Further, this area of research may also help identify strategies (e.g., mental imagery of exercise) to facilitate memory performance. Alternatively, if these behaviors have different effects on memory, then there may be distinct mechanisms through which actual and imagined exercise influence memory. 

## 2. Methods

### 2.1. Study Design

A within-subject randomized controlled experiment was employed. Participants completed two exercise-related visits (i.e., actual exercise and mental imagery of exercise), in a counterbalanced order. Prior to these two exercise-related visits, the participants completed a familiarization visit. All visits occurred around the same time of day and within 48–72 h of each other. This study was approved by the ethics committee at the University of Mississippi (#19-040). Participants provided written consent prior to participation. 

### 2.2. General Protocol for Visits

Details for the three visits are as follows. 

First Visit: Familiarization

- Participants were familiarized with the exercise protocol, for both the actual exercise engagement and mental imagery of exercise task. This involved showing the participants where the actual exercise would occur, which was along a straight line inside an indoor racquetball court.
- Participants performed several sprints along this pathway to allow them to be familiarized with the subsequent visits (actual vs. mental imagery of acute exercise). After they performed these sprints, which involved jogging from one end of the court to the other and touching the wall on the opposite side once they reached it, they engaged in a familiarization session of imagined exercise. That is, they sat down and, with their eyes closed, imagined themselves running to the other side of the court, touching the wall, and running back.

Actual Acute Exercise Visit:

- For 10 min, participants engaged in an acute exercise bout that involved running sprints in an indoor racquetball court (20 s sprint, followed by 60 s of walking; repeated until 10 min had elapsed).
- After a 2 min slow walk back to the laboratory, participants engaged in 5 min of seated rest.
- Participants engaged in a paired-associate learning (PAL) task, a 30 s arithmetic distraction task, and then immediate recall of the PAL.
- Participants performed a treasure-hunt task (what-where-when) and then a delayed recall of the PAL.
- Participants then left the laboratory.

Mental Imagery of Acute Exercise Visit:

- For 10 min, participants engaged in mental imagery of running sprints (20 s sprint, followed by 60 s of walking; repeated until 10 min had elapsed). The mental-imagery session occurred while participants were seated in the racquetball court.
- After a 2 min slow walk to the laboratory, participants engaged in 5 min of seated rest.
- Participants engaged in a paired-associate learning (PAL) task, a 30 s arithmetic distraction task, and immediate recall of the PAL.
- Participants performed a treasure-hunt task (what-where-when) and then delayed recall of the PAL.

## 3. Participants

The study included 24 participants. Recruitment occurred via a convenience-based, non-probability sampling approach (classroom announcement and word-of-mouth). Participants were eligible if they were undergraduate or graduate students between the ages of 18 and 40 years. Notably, those who participated were 19–24 years of age.

Additionally, participants were excluded if they: self-reported as a daily smoker [14,15]; self-reported as being pregnant [16]; exercised within 5 h of testing [17]; consumed caffeine within three hours of testing [18]; had a concussion or head trauma within the past 30 days [19]; took marijuana within the past 30 days [20]; were considered a daily alcohol user (>30 drinks/month for women; >60 drinks/month for men) or consumed alcohol on the day of testing [21].

Notably, no recruited participants ended up being excluded due to these criteria. 

### 3.1. Exercise Assessment

The actual exercise bout was adapted from other related studies [7,8]. Participants engaged in 10 min of repeated interval runs. This involved jogging for 20 s on a straight line (at a self-selected running speed), followed by slow walking for 60 s. This cycle was repeated until 10 min had elapsed.

The mental imagery of acute exercise was practiced in a seated position. Participants completed the same protocol as the actual exercise but did so via mental imagery (eyes closed). They were instructed to think back to their actual-exercise visit (or familiarization visit) and, for the mental-imagery protocol, imagine themselves engaging in the running and walking intervals. A researcher prompted them to start imagining running/walking every 20 s. This procedure also lasted 10 min.

As a manipulation check of the mental-imagery protocol, after the mental-imagery session, participants completed two survey questions, both with response options (1) strongly disagree, (2) disagree, (3) neutral, (4) agree, and (5) strongly agree. The first question was, “During the 10-min imagination session, I was able to effectively imagine myself jogging and walking.” The second question was, “During the 10-min imagination session, my mind frequently wandered, and I became distracted.”

### 3.2. Memory Assessment

For the paired-associate learning (PAL) memory assessment, participants completed a paired-associate learning task, as this task of cued recall has shown evidence of being hippocampal-dependent [22,23]. Ten word pairs (two-syllable) from the Medical Research Council Psycholinguistic Database were used, with each word having an imageability score between 414 and 486, to reduce variability on this parameter, which can influence cued-recall performance [24]. Participants viewed each word pair on a computer screen for 5-sec. After the 10th word pair was presented, participants verbally completed several simple arithmetic problems for 30 s. After this 30 s distraction period, an immediate cued-recall test was performed. For this, the first word from each pair was presented, and the participant attempted to recall the second word from the pair. Both a short-term and long-term cued-recall assessment occurred. In between the short- and long-term memory assessments, participants completed the THT (described below). Separate paired-associate learning tasks occurred for the two visits.

The treasure-hunt task (THT) is a computerized task assessing what-where-when memory, taking approximately 10 min to complete. Details of this THT were discussed elsewhere [25,26]. In brief, this task involves ‘hiding’ items in various scenes and then later indicating what items were hidden, where, and on what occasion. This requires the integration of item, location, and temporal memory into a single coherent representation (what-where-when memory, WWW). Participants are also assessed for their memory for the individual components (what, where, and when) without requirement for integration. Internal consistency for these tasks was previously demonstrated (ICCs > 0.7) [26]. The outcome variables assessed included an absolute WWW score (in which the location of the correct object for the correct time is identified exactly) and the proportion of correct responses for the separate what, where, and when subtasks. This study used the ‘hard’ difficulty version of the task, involving 24 unique items. A separate (but of equal difficulty) THT was employed for the two visits.

### 3.3. Statistical Analysis

All statistical analyses were computed in JASP (v. 0.10, Amsterdam, The Netherlands). Bayesian analyses were conducted. For the THT, a one-factor repeated-measures ANOVA was employed for each of the THT metrics (i.e., overall integration and what-where-when aspects). For the paired-associative learning task, a 2 (condition) × 2 (time; immediate vs. delay) factor repeated-measures ANOVA was employed. Main effects for time (immediate and delay), main effects for condition (actual exercise vs. imagined exercise), and time by condition interactions were evaluated. The Bayes Factor for the interaction analyses was calculated from the ratio of the interaction model (Factor 1 + Factor 2 + Factor 1×Factor) over the main effects model (Factor 1 + Factor 2). From the Bayesian analyses, Bayes Factors (BFs) are reported (Lee and Wagenmakers [27], as cited by Wagenmakers et al. [28]). 

## 4. Results

Table 1 displays the characteristics of the sample. Participants, on average, were 21.5 years of age and were predominately female (54.2%) and non-Hispanic white (83.3%). 

Table 2 displays the physiological (heart rate) response of the two conditions. In the mental-imagery-exercise condition, heart rates stayed in the upper 70 s and low 80 s (bpm). In the actual exercise session, heart rates increased from 83.4 to 151 bpm. 

Regarding the mental-imagery manipulation check, the first question was, “During the 10-min imagination session, I was able to effectively imagine myself jogging and walking.” The second question was, “During the 10-min imagination session, my mind frequently wandered, and I became distracted.” The mean (SD) for the first question was 4.0 (0.5), which corresponds with an “agree” response. The mean (SD) for the second question was 3.3 (0.8), which corresponds with a “neutral” response. Bivariate correlation analyses were computed to evaluate the association between these two survey questions and the memory performances from the mental-imagery visit. All associations were weak. The range in the associations between the first survey item and the different memory outcomes was r = –0.26 to r = 0.09. The range in the associations between the second survey item and the different memory outcomes was r = –0.27 to r = 0.15. Thus, these finding suggest that any potential mind-wandering effects likely did not alter the effects of the mental-imagery session on memory performance. 

Table 3 displays the memory results. For the PAL task, there was no evidence of a main effect for time (BF_01_ = 0.92), but there was moderate evidence in favor of the null hypothesis for a main effect for condition (BF_01_ = 2.85) and time by condition interaction (BF_01_ = 3.30). 

Similarly, and for the THT, there was moderate evidence in favor of the null hypothesis for overall (WWW) memory integration (BF_01_ = 3.37), what-loop (BF_01_ = 2.34), where-loop (BF_01_ = 3.45), and when-loop (BF_01_ = 3.46).

## 5. Discussion

This experiment set out to determine whether memory function is differentially influenced by acute exercise vs. mental imagery of acute exercise. The motivation for this experiment was from prior research demonstrating that (1) mental imagery of exercise can enhance sport performance and cognitive attributes related to support performance, and (2) memory-related brain structures are activated during both actual exercise engagement and mental imagery of acute exercise. This aligns with the embodied cognition framework, suggesting an interdependence between perception, cognition, and movement [13]. The main finding from this experiment was that there is moderate evidence to support the null hypothesis (i.e., that there is no difference in memory when comparing acute-exercise engagement to mental imagery of acute exercise). 

Thus, these findings suggest that mental imagery of acute exercise may elicit a similar effect on memory function when compared to actual engagement in acute exercise. We can interpret these findings in several ways. It is possible that actual exercise and mental imagery of exercise have the same (beneficial) effect on memory. Alternatively, one could argue that this similar effect may be driven by the actual exercise session not being effective in eliciting improvements in memory. This latter point could be addressed by including a control group that does not exercise and does not engage in mental imagery. This, however, was not the focus of the present study. That is, the purpose of this study was to determine whether acute-exercise engagement has a differential effect on memory when compared to mental imagery. Numerous studies have already compared acute exercise to a non-exercise, non-imagery control group and have shown that acute exercise is beneficial in enhancing memory when compared to this control scenario [4,29,30].

Future work should continue to explore this novel paradigm. If such work confirms that mental imagery of acute exercise has a similar effect to actual exercise on memory, then this may help spawn the development of an interesting set of experiments to follow. For example, it would be interesting to evaluate whether acute exercise coupled with mental engagement during exercise has an additive effect on memory. Alternatively, it would be interesting to see if mental imagery prior to actual engagement in exercise has a priming effect on memory. 

Limitations of this study include the relatively small, homogenous sample. We were also not able to effectively prevent participants’ minds from wandering during the mental-imagery session, which is not surprising, as 10 min may be perceived as a long time to engage in mental imagery. This issue, however, was likely not driving our findings. That is, participants were still able to effectively engage in mental imagery. Further, if mind-wandering was detrimental to our findings, then we would have expected a differential effect of acute exercise vs. mental imagery on memory (i.e., increased mind-wandering resulting in a reduced effectiveness of the mental-imagery session and, in turn, having less of an effect on memory). Past work has highlighted individual differences in mental-imagery effectiveness [13]. Future research should explore ways to maximize compliance with the mental-imagery session. This could be done through a more extensive familiarization (i.e., multiple sessions) of mental imagery or reducing the length of the mental-imagery session. The latter, however, may compromise the effectiveness of the actual exercise session, as it may be important to time-match the actual-exercise and mental-imagery sessions. Further, the present study, of course, cannot confirm that similar brain regions were activated during the acute exercise and mental imagery sessions. As such, when feasible, future work should consider exploring this. As stated, this is an area of research that is ripe for investigation. In addition to our study’s limitations and recommendations for future research, we also acknowledge the strengths of this study. This includes the experimental design, study novelty, manipulation check of the mental imagery session, contextual congruence between the two sessions (i.e., both occurring in the same environment), and a comprehensive assessment of memory. 

## 6. Conclusions

In conclusion, this experiment provides moderate evidence in support of the null hypothesis. That is, there was moderate evidence to support a non-differential effect of acute exercise and mental imagery of acute exercise on memory function. 

## Figures and Tables

**Table 1:** Characteristics of the sample.

Variable	Mean (SD)
Age, mean years	21.5 (1.2)
Gender, % Female	54.2
Race-Ethnicity, % White	83.3
BMI, mean kg/m^2^	25.4 (4.0)
MVPA, mean min/week	167.9 (115.5)

MVPA, moderate-to-vigorous physical activity, as assessed from the Physical Activity Vitals Sign survey.

**Table 2:** Heart rate responses to the experimental manipulation.

Condition	Mean BPM
**Actual Exercise**	
Rest	83.4 (8.4)
Midpoint	150.7 (19.8)
Endpoint	151.1 (18.7)
**Mental Imagery of Exercise**	
Rest	77.3 (11.4)
Midpoint	78.5 (13.7)
Endpoint	84.7 (13.7)

BPM, beats per minute.

**Table 3:** Memory outcomes across the experimental conditions.

PAL, Mean #	Actual Exercise	Mental Imagery of Exercise
Immediate	3.9 (2.5)	3.6 (1.8)
Delayed	3.3 (2.6)	3.0 (1.9)
**WWW, mean %**		
Overall	59.9 (28.2)	61.5 (21.3)
What	96.3 (9.5)	98.1 (2.4)
Where	83.6 (8.8)	84.3 (13.9)
When	89.5 (8.2)	90.0 (9.7)

PAL, paired-associative learning; WWW, what-where-when; #, Number.

## References

[1] Jones L., Stuth G. (1998). The uses of mental imagery in athletics: An overview. Appl. Prev. Psychol..

[2] Loprinzi P.P., Herod S.S., Cardinal B.B., Noakes T.T. (2013). Physical activity and the brain: A review of this dynamic, bi-directional relationship. Brain Res..

[3] Sng E., Frith E., Loprinzi P.P. (2017). Temporal Effects of Acute Walking Exercise on Learning and Memory Function. Am. J. Health Promot..

[4] Frith E., Sng E., Loprinzi P.P. (2017). Randomized controlled trial evaluating the temporal effects of high-intensity exercise on learning, short-term and long-term memory, and prospective memory. Eur. J. Neurosci..

[5] Haynes J.T., Frith E., Sng E., Loprinzi P.P. (2018). Experimental Effects of Acute Exercise on Episodic Memory Function: Considerations for the Timing of Exercise. Psychol. Rep..

[6] Loprinzi P.P. (2018). Intensity-specific effects of acute exercise on human memory function: Considerations for the timing of exercise and the type of memory. Health Promot. Perspect..

[7] Jahn K., Deutschlander A., Stephan T., Strupp M., Wiesmann M., Brandt T. (2004). Brain activation patterns during imagined stance and locomotion in functional magnetic resonance imaging. Neuroimage.

[8] La Fougere C., Zwergal A., Rominger A., Förster S., Fesl G., Dieterich M., Brandt T., Strupp M., Bartenstein P., Jahn K. (2010). Real versus imagined locomotion: A [18F]-FDG PET-fMRI comparison. Neuroimage.

[9] Fliessbach K., Trautner P., Quesada C.C., Elger C.C., Weber B. (2007). Cerebellar contributions to episodic memory encoding as revealed by fMRI. Neuroimage.

[10] Aminoff E.E., Kveraga K., Bar M. (2013). The role of the parahippocampal cortex in cognition. Trends Cogn. Sci..

[11] Loprinzi P.P. (2019). The effects of physical exercise on parahippocampal function. Physiol. Int..

[12] Pearson J. (2019). The human imagination: The cognitive neuroscience of visual mental imagery. Nat. Rev. Neurosci..

[13] Palmiero M., Piccardi L., Giancola M., Nori R., D’Amico S., Olivetti Belardinelli M. (2019). The format of mental imagery: From a critical review to an integrated embodied representation approach. Cogn. Process..

[14] Jubelt L.L., Barr R.R., Goff D.D., Logvinenko T., Weiss A.A., Evins A.A. (2008). Effects of transdermal nicotine on episodic memory in non-smokers with and without schizophrenia. Psychopharmacology.

[15] Klaming R., Annese J., Veltman D.D., Comijs H.H. (2016). Episodic memory function is affected by lifestyle factors: A 14-year follow-up study in an elderly population. Neuropsychol. Dev. Cogn. B Aging Neuropsychol. Cogn..

[16] Henry J.J., Rendell P.P. (2007). A review of the impact of pregnancy on memory function. J. Clin. Exp. Neuropsychol..

[17] Labban J.J., Etnier J.J. (2011). Effects of acute exercise on long-term memory. Res. Q. Exerc. Sport..

[18] Sherman S.S., Buckley T.T., Baena E., Ryan L. (2016). Caffeine Enhances Memory Performance in Young Adults during Their Non-optimal Time of Day. Front. Psychol..

[19] Wammes J.J., Good T.T., Fernandes M.M. (2017). Autobiographical and episodic memory deficits in mild traumatic brain injury. Brain Cogn..

[20] Hindocha C., Freeman T.T., Xia J.J., Shaban N.D.C., Curran H.H. (2017). Acute memory and psychotomimetic effects of cannabis and tobacco both ‘joint’ and individually: A placebo-controlled trial. Psychol. Med..

[21] Le Berre A.A., Fama R., Sullivan E.E. (2017). Executive Functions, Memory, and Social Cognitive Deficits and Recovery in Chronic Alcoholism: A Critical Review to Inform Future Research. Alcohol. Clin. Exp. Res..

[22] Caplan J.J., Madan C.C. (2016). Word Imageability Enhances Association-memory by Increasing Hippocampal Engagement. J. Cogn. Neurosci..

[23] Mayes A., Montaldi D., Migo E. (2007). Associative memory and the medial temporal lobes. Trends Cogn. Sci..

[24] Paivio A. (1969). Mental imagery in associative learning and memory. Psychol. Rev..

[25] Cheke L.L. (2016). What-where-when memory and encoding strategies in healthy aging. Learn. Mem..

[26] Cheke L.L., Simons J.J., Clayton N.N. (2016). Higher body mass index is associated with episodic memory deficits in young adults. Q. J. Exp. Psychol..

[27] Lee M.M., Wagenmakers E.E. (2013). Bayesian Cognitive Modeling: A practical guide.

[28] Wagenmakers E.J., Love J., Marsman M., Jamil T., Ly A., Verhagen J., Selker R., Gronau Q.F., Dropmann D., Meerhoff F. (2018). Bayesian inference for psychology. Part II: Example applications with JASP. Psychon. Bull. Rev..

[29] Loprinzi P.P., Blough J., Crawford L., Ryu S., Zou L., Li H. (2019). The temporal effects of acute exercise on episodic memory function: Systematic review with meta-analysis. Brain Sci..

[30] Winter B., Breitenstein C., Mooren F.C., Voelker K., Fobker M., Lechtermann A., Krueger K., Fromme A., Korsukewitz C., Knecht S. (2007). High impact running improves learning. Neurobiol. Learn. Mem..

